# Functional impact for indication and access to physical therapy after hospital discharge due to COVID-19

**DOI:** 10.1016/j.clinsp.2025.100807

**Published:** 2025-10-16

**Authors:** Vivian Cintra Sousa, Fabio Cavalcanti Freitas, Erika Christina Gouveia e Silva, Nayara Oliveira Santos, Daniella de Melo, Sara Cristina Aparecida da Silva, Debora Stripari Schujmann Nogueira, Carolina Fu, Caroline Gil de Godoy, José Eduardo Pompeu, Ana Carolina Basso Schmitt

**Affiliations:** a Occupational Therapy Department, Faculdade de Medicina da Universidade de São Paulo, São Paulo, SP, Brazil; b Physiotherapy Department, Universidade Cruzeiro do Sul, Guarulhos, SP, Brazil

**Keywords:** Covid-19, Rehabilitation, Functional status, Health services accessibility, Patient discharge

## Abstract

**Introduction:**

After hospitalization for COVID-19, patients may present impairment in functionality and require physical therapy after hospital discharge for functional recovery.

**Objective:**

To understand the association between Covid-19 functional impacts and physical therapy indication and access 30 days and 1 year after hospital discharge of severely and critically ill patients.

**Methods:**

Cross-sectional study with two assessments: 30 days and one year after hospital discharge, in individuals ≥ 18 years of age, admitted to a referral hospital in São Paulo between June 2020 and July 2021. A convenience sample of 345 patients was used. The Poisson test was used to estimate the prevalence ratio for the association between Covid-19 functional impacts and physical therapy indication and access, with ≤ 0.05 considered significant.

**Results:**

Of the 185 patients included, 67 % (n: 104) were indicated for physical therapy and the majority (53 %; n:79) could not access it 30 days after hospital discharge. Post-Covid-19 functional limitations were associated with physical therapy indication (PR: 1.69; 95 %CI 1.1–2.5) and impairment in basic activities of daily living (BADLs) with access 30 days (PR: 1.81; 95 %CI 1.2 -2.6) and 1 year after discharge (PR: 1.70; 95 %CI 1.2–2.3). Physical therapy indication was significant, with a 4.07 and 2.06 likelihood of access 30 days and 1 year after discharge.

**Conclusion:**

Despite the lack of functional criteria at discharge, patients with poor functional performance perceived the need for physical therapy indication and referral. Indication was essential for access to physiotherapy within the healthcare network.

## Highlights

• Functional impacts after hospitalization for Covid-19.
• Continuity of physical therapy care after hospitalization for Covid-19.
• Criteria for physical therapy after Covid-19.
• Functionality as a criterion for physical therapy indication after hospital discharge.

## Introduction

Coronavirus disease 2019 (Covid-19) is an infectious disease caused by the SARS-CoV-2virus,^1^ which was first identified in China in December 2019^2^ In Brazil, the first case was confirmed in February 2020, with >37 million cases and almost 705,000 deaths recorded by early November 2023^3^ Its presentation can be symptomatic or asymptomatic^2^ This multi-organ disorder affects the respiratory, cardiovascular, gastrointestinal, neurological, and musculoskeletal systems^4^

About 15 and 5 % of infected people develop the severe and critical forms of the disease, respectively, with complications including respiratory failure, acute respiratory distress syndrome (ARDS), sepsis, septic shock, thromboembolism, and multiple organ failure^2^^,^^5^^,^^6^ Severe and critical patients require hospitalization and may experience complications linked to length of stay, bed rest, and use of sedatives, among others, leading to functional impairment. This condition is known as post-intensive care syndrome (PICS) and can persist up to 5 years after hospital discharge^2^^,^^7^^,^^8^

These post-hospitalization complications can affect bodily functions and structures, limiting performance in basic (BADLs) and instrumental activities of daily living (IADL), the former related to personal care and mobility and the latter to the ability to interact with the environment^9^

As a result, the Pan American Health Organization highlights the need to create and adapt public or private referral services to rehabilitate individuals after Covid-19, promoting the continuity of care and rehabilitation after discharge^10^ However, for this to occur, physiotherapy must be indicated and patients must have access to the service, that is, "the ability to reach and receive appropriate health services in situations where a need for care is perceived"^11^

Despite the obvious need for physical therapy after hospital discharge due to Covid-19 and PICS sequelae,^12^^,^^13^ the functional criteria for referral at discharge are unclear, falling to healthcare professionals to recommend physical therapy. Thus, understanding functional impacts and criteria for referral after hospital discharge is essential to ensure efficient and timely access to health and rehabilitation services for the continuity of comprehensive health care.

Thus, the present study aimed to understand the association between Covid-19 functional impacts and physical therapy indication and access 30 days and one year after hospital discharge of severely and critically ill patients.

## Methods

### Study design

This cross-sectional times series study involved two assessments, conducted 30 days and one year after hospital discharge.

### Setting

Individuals ≥ 18 years of age of both sexes, diagnosed with COVID-19 and admitted to a referral hospital for severe cases in São Paulo state, Brazil, between June 2020 and July 2021 were included. The study was approved by the Ethics Committee of the Clinics Hospital of the University of Sao Paulo’s School of Medicine - HC-FMUSP (CAEE: 34,115,720.5.0000.0068), and all participants signed an Informed Consent Form (ICF).

### Participants

Inclusion criteria were hemodynamically stable individuals with preserved or corrected visual and auditory acuity, capable of understanding simple commands. Excluded were patients unavailable on the scheduled assessment days, those with cognitive impairments that prevented them from understanding the instruments applied, and unstable clinical parameters on assessment days, as well as duplicate medical records, and missing essential data.

### Study size

Since the study was conducted during the critical period of the Covid-19 pandemic, when healthcare services were overwhelmed, a convenience sample of 345 patients was used. Patients were contacted by telephone and invited to participate in the study, with assessments conducted by phone and in person 30 days and 1 year after discharge. A total of 185 people agreed to take part in the study. Statistical power for physical therapy indication was 98.43 %, 10.80 % for access to physical therapy 30 days after discharge, and 97.65 % after one year, considering a 95 % confidence interval.

### Variables

The dependent variables were i. *physical therapy indication*, ii. *access to physical therapy 30 days after discharge*, and iii. *access to physical therapy one year after discharge*. Physical therapy was considered indicated when participants reported that it had been recommended or that they needed to undergo physical therapy. Access to physical therapy was considered positive when they cited the location where they were receiving physiotherapy, both 30 days and 1 year after hospital discharge.

The independent variables were age, sex, race, marital status, schooling level, income, length of hospital stay, intensive care unit (ICU) admission, invasive mechanical ventilation, and the reason for physical therapy indication. Functional impacts were measured using different instruments, based on changes in the following variables: post-Covid-19 functional impacts, using the Post-Covid-19 Functional Status (PCFS) scale^14^; IADLs, via the Lawton scale^15^; BADLs, according to the Katz scale^16^ and Barthel Index^17^; frailty, with the Clinical Frailty Scale (CFS)^18^; sarcopenia, by Sarcopenia Risk Screening (SARC-F)^19^; cognition, using the 10-Point Cognitive Screener (10-CS)^20^; perceived fear of falling, by the Falls Efficacy Scale – International (FES-I)^21^; muscle fatigue, in accordance with the Functional Assessment of Chronic Illness Therapy (FACIT)^22^ scale; mobility, via Life Space Assessment (LSA)^23^; balance, by the BESTest Brief^24^; functional capacity, with the Sit-to-Stand Test (5 times)^25^; handgrip strength, using handheld dynamometry^26^; respiratory function, via spirometry^27^; and functional mobility, by the Timed Up and Go (TUG) test with a G-walk sensor^28^

Data were collected and stored using Research Electronic Data Capture (RedCap) software.

### Statistical methods

Data normality was tested in Stata 14 and considered non-parametric. Descriptive measures of central tendency and dispersion, as well as percentages, were used. The prevalence ratio was measured via Poisson distribution, considering the association between Covid-19 functional impacts (post-Covid-19 functional impact, instrumental activities of daily living, basic activities of daily living, frailty, sarcopenia, cognition, perception of fear of falling, muscle fatigue, mobility, balance,functional capacity, handgrip strength, respiratory function, functional mobility) and i. physical therapy indication,and ii. access 30 days and iii. one year after discharge. Significance was set at *p* ≤ 0.05.

## Results

### Participants

**Fig. 1:**
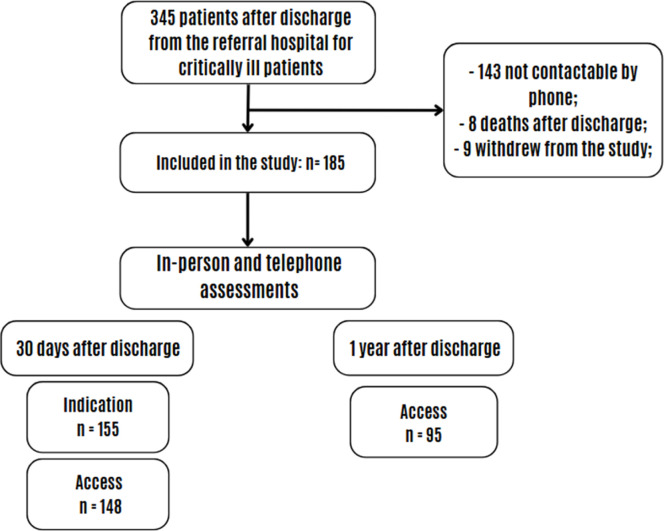
Study flowchart. n, number of patients.

Of the 185 Covid-19 patients included in the study, 155 participated 30 days after hospital discharge and 95 one year post-discharge, as shown in Fig. 1.

### Descriptive data

Table 1 shows the demographic, clinical, and care characteristics according to physical therapy indication and access. Participants’ median age was 59 (49–67) years, 56 % (*n* = 103) were male, and 49.7 % (*n* = 90) white. The median length of hospital stay was 17.5 (10–30) days, and exhibited a significant association with physical therapy indication 30 days after discharge (*p* = 0.046). A significant association was also observed between age (*p* = 0.029) and sex (*p* = 0.001) and physical therapy access one year after discharge. Most patients (83.5 %) were admitted to the intensive care unit, but there was no association with physical therapy indication or access.

**Table 1:** Demographic, clinical, and care characteristics according to physical therapy indication and access 30 days and one year post-discharge from hospitalization for Covid-19.

**Demographic, clinical and care characteristics**	**Indication of physiotherapy 30-days**			**Access to physiotherapy after 30-days**			**Access to physiotherapy after 1-year**		
	**No**	**Yes**	**p-valor^a^**	**No**	**Yes**	**p-valor^a^**	**No**	**Yes**	**p-valor^a^**
	**n ( %)**	**n ( %)**		**n ( %)**	**n ( %)**		**n ( %)**	**n ( %)**	
**Age**	51 (32.9)	104 (67.1)	0.723	79 (53.4)	69 (46.6)	0.284	28 (29.5)	67 (70.5)	0.029
Adults up to 59	29 (34.1)	56 (65.9)		40 (49.4)	41 (50.6)		21 (38.2)	34 (61.8)	
Seniors over 60	22 (31.4)	48 (68.6)		39 (58.2)	28 (41.8)		7 (17.5)	33 (82.5)	
**Sex**	51(32.9)	104 (67.1)	0.418	79 (53.4)	69 (46.6)	0.816	28 (29.5)	67 (70.5)	0.001
Male	30 (35.7)	54 (64.3)		42 (52.5)	38 (47.5)		23 (44.2)	29 (55.8)	
Female	21 (29.6)	50 (40.4)		37 (54.4)	31 (45.6)		5 (11.6)	38 (88.4)	
**Race**	51 (32.9)	104 (67.1)	0.200	79 (53.7)	68 (46.3)	0.229	28 (29.8)	66 (70.2)	0.520
White	22 (27.8)	57 (72.2)		36 (48.6)	38 (51.4)		15 (34.1)	29 (65.9)	
Black/Brown	29 (39.2)	45 (60.8)		41 (57.7)	30 (42.3)		12 (25.0)	36 (75.0)	
Others (yellow/indigenous)	0 (0.0)	2 (100.0)		2 (100.0)	0 (0)		1 (50.0)	1 (50.0)	
**Marital status**	51 (32.9)	104 (67.1)	0.976	79 (53.4)	69 (46.6)	0.474	28 (29.5)	67 (70.5)	0.198
In a relationship	19 (32.8)	39 (67.2)		32(57.1)	24 (42.9)		7 (21.2)	26 (78.8)	
Single	32 (33.0)	65 (67.0)		47 (51.1)	45 (48.9)		21 (33.9)	41 (66.1)	
**Schooling**	51 (33.3)	102 (66.7)	0.226	78 (53.4)	68 (46.6)	0.079	28 (29.5)	67 (70.5)	0.996
Basic	28 (40.6)	41 (59.4)		40 (60.6)	26 (39.4)		11 (28.9)	27 (71.1)	
Middle school	15 (27.3)	40 (72.7)		29 (53.7)	25 (46.3)		11 (29.7)	26 (70.3)	
Higher education	8 (27.6)	21 (72.4)		9 (34.6)	17(65.4)		6 (30.0)	14 (70.0)	
**Length of hospital stay**	27 (35.5)	49 (64.5)	0.046	34 (49.3)	35 (50.7)	0.641	13 (32.5)	27 (67.5)	0.706
Up to 15 days	12 (42.9)	16 (57.1)		15 (55.7)	12 (44.4)		5 (38.5)	8 (61.5)	
Up to 30 days	13 (43.3)	17 (56.7)		12 (48.0)	13 (52.0)		4 (25.0)	12 (75.0)	
Up to 68 day	2 (11.1)	16 (88.9)		7 (41.2)	10 (58.2)		4 (36.4)	7 (63.6)	
**Intensive Care Unit**	51 (32.9)	104 (67.1)	0.650	79 (53.4)	69 (46.6)	0.876	28 (29.8)	66 (70.2)	0.846
No	6 (28.6)	15 (71.4)		11 (55.0)	9 (45.0)		3 (27.3)	8 (72.8)	
Yes	45 (33.6)	89 (66.4)		68 (53.1)	60 (46.9)		25 (30.1)	58 (69.9)	
**Invasive ventilation**	50 (33.1)	101 (66.9)	0.114	76 (52.8)	68 (47.2)	0.172	28 (29.8)	66 (70.2)	0.413
No	24 (40.7)	35 (59.3)		33 (60.0)	22 (40.0)		10 (35.7)	18 (64.3)	
Yes	26 (28.3)	66 (71.4)		43 (48.3)	46 (51.9)		18 (27.3)	48 (72.7)	
**Income**	28 (34.6)	53 (65.4)	0.156	35 (47.3)	39 (52.7)	0.292	12 (28.6)	30 (71.4)	0.823
up toR$2000,00	12 (44.4)	15 (55.6)		16 (59.3)	11 (40.7)		4 (28.6)	10 (71.4)	
R$2000,000 ‒ R$5000,00	11 (25.0)	33 (75.0)		16 (40.0)	24 (60.0)		6 (26.1)	17 (73.9)	
R$5000,000 ‒ R$10,000,00	5 (50.0)	5 (50.0)		3 (42.9)	4 (57.1)		2 (40.0)	3 (60.0)	

n, number of patients. a Poisson; R$, Brazilian currency.

### Main results

**Fig. 2:**
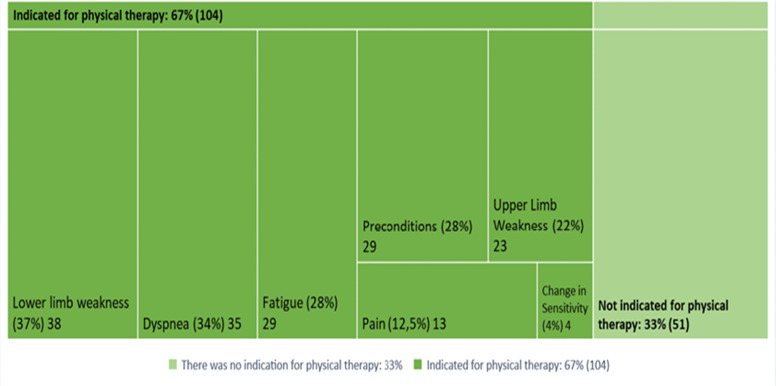
Patient perception regarding why they were indicated for post-discharge physical therapy after hospitalization for Covid-19.

**Fig. 3:**
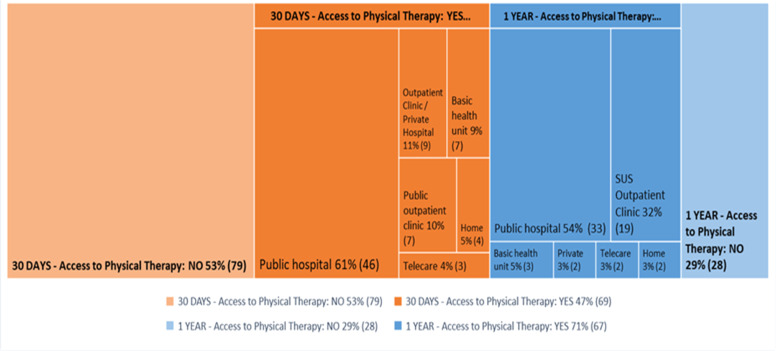
. Distribution of places for physical therapy after 30-days and one year. Figure 3. Distribution of physical therapy locations 30 days and one year post-discharge.

Figs. 2 and Fig. 3 show that of the 155 people who responded to the question regarding indication, 67.1 % (*n* = 104) were indicated for post-discharge physiotherapy. The main reasons, according to patient perception, were lower limb muscle weakness in 37 % (*n* = 38), dyspnea in 34 % (*n* = 35), fatigue in 28 % (*n* = 29) and pre-Covid-19 conditions in 28 % (*n* = 29). Of the 148 individuals who answered the question regarding access, 46.6 % (*n* = 69) had access to physiotherapy 30 days after discharge, with 60.5 % (*n* = 46) receiving it at the hospital itself. Of the 95 people that responded regarding access one year after discharge, 70.5 % (*n* = 67) had access to physiotherapy, 54.1 % (*n* = 33) of whome received it at the hospital where they were treated.

Table 2 shows the relationship between physical therapy indication and access. Almost 60 % of those indicated for physical therapy at discharge had access to it within 30 days (PR: 4.07; 95 %CI 1.9 - 8.6), and 80 % within 1 year (PR: 2.06; 95 %CI 1.2 - 3.4).

**Table 2:** Bivariate analysis of physical therapy access 30 days and one year post-discharge after hospitalization for Covid-19, according to physiotherapy indication.

Physical therapy access	Physical therapy indication after hospital discharge		
	Yes, n ( %)	RP (CI 95 %)	p-valor^a^
Physical therapy access 30 days after discharge	62 (59.6)	4.07 (1.9‒8.6)	0.000
Physical therapy access 1 year after discharge	55 (80.9)	2.06 (1.2‒3.4)	0.007

n, number of patients; PR, prevalence ratio; CI, confidende interval.

a Poisson.

There were a total of 270 results indicating some degree of functional impact, albeit with no indication for physical therapy. Some functional impacts were related to physical therapy indication 30 days after discharge, with the most significant being post-Covid-19 functional limitation (PR: 1.69; 95 %CI 1.1 - 2.5); IADL impairment (PR: 1.52; 95 %CI 1.3 - 1.7); dependence in BADLs (PR: 1.52; 95 %CI 1.1 - 1.9); significant concern about falls (PR: 1.52; 95 %CI 1.1 - 2.0); increased frailty (PR: 1.50; 95 %CI 1.0 - 2.2); greater risk of sarcopenia (PR: 1.37; 95 %CI 1.1 - 1.6); dependence when walking (PR: 1.33; 95 %CI 1.0 - 1.6) and severe fatigue (PR: 0.7; 95 %CI 0.6 - 0.9) (Table 3).

**Table 3:** Bivariate analysis of functional impact, according to physical therapy indication and post-discharge access 30 days and one year after hospitalization for COVID-19.

**Functional impact**	**Indication of physiotherapy 30 days**			**Access to physiotherapy after 30 days**			**Access to physiotherapy after 1 year**		
	**Yes**	**RP (95 % CI)**	**p-valor^a^**	**Yes**	RP (95 % CIy)y	**p-valor^a^**	**Yes**	**RP (95 % CI)**	**p-valor^a^**
	**n ( %)**			**n ( %)**			**n ( %)**		
**BADLs (Katz)**									
Full Function	80 (90.9)	1		51 (86.5)	1		17 (94.4)	1	
With commitment	8 (9.1)	1.24 (0.8‒1.7)	0.212	8 (13.5)	1.81 (1.2‒2.6)	**0.002**	1 (5.6)	1.70 (1.2‒2.3)	**0.001**
**BADLs (Barthel)**					1.81 (1.2‒2.6)				
Independent	39 (40.6)	1		30 (46.9)	1		23 (51.1)	1	
Dependent	57 (59.4)	1.52 (1.1‒1.9)	**0.001**	34 (53.1)	1.11 (0.7‒1.6)	0.549	22 (48.9)	0.69 (0.4 1.1)	0.164
**IADLs**									
Independent	98 (94.2)	1		65 (94.2)	1		67 (100.0)		
Dependent	6 (5.8)	1.52 (1.3‒1.7)	**0.000**	4 (5.8)	1.23 (0.6‒2.4)	0.529			
**Ambulation**									
Independent	41 (40.2)	1		28 (41.2)	1		19 (28.4)	1	
Dependent	61 (59.8)	1.33 (1.0‒1.6)	**0.015**	40 (58.8)	1.19 (0.8‒1.7)	0.331	48 (71.6)	1.56 (1.1‒2.1)	**0.008**
**Post Covid-19 Functionality**									
No limitations	13(12.5)	1		8 (11.6)	1		13 (22.0)	1	
With limitations	91 (87.5)	1.69 (1.1‒2.5)	**0.015**	61 (88.4)	1.63 (0.8‒3.0)	0.110	46 (78.0)	1.42 (0.9‒2.1)	0.087
**Fraily**									
Not fragil	14 (14.4)	1		15 (23.1)	1		16 (27.1)	1	
Frail	83 (85.6)	1.50 (1.0‒2.2)	**0.045**	50 (76.9)	0.90 (0.6‒1.3)	0.652	43 (72.9)	0.72 (0.4‒1.0)	0.096
**Concern about Falls**									
Low	29 (27.9)	1		21 (30.4)	1		28 (47.5)	1	
Moderate	34 (32.7)	1.34 (0.9‒1.8)	0.062	24 (34.8)	1.24 (0.8‒1.9)	0.321	15 (25.4)	1.63 (1.2‒2.2)	**0.002**
High	41 (39.4)	1.52 (1.1‒2.0)	**0.004**	24 (34.8)	1.12 (0.7‒1.7)	0.611	16 (27.1)	1.65 (1.2‒2.2)	**0.001**
**Risk of falling**									
No	37 (52.9)	1		22 (48.9)	1		40 (81.6)	1	
Yes	33 (47.1)	1.15 (0.8‒1.4)	0.271	23(51.1)	1.23(0.8‒1.8)	0.332	9 (18.4)	0.07(0.4 ‒1.1)	0.183
**Sarcopenia (Sitting and Standing)**									
Non-sarcopenia	30 (34.1)	1		23 (39.0)	1		26 (59.1)	1	
Sarcopenia	58 (65.9)	1.21 (0.9‒1.5)	0.151	36 (61.0)	0.9 (0.6‒1.3)	0.630	18 (40.9)	0.97 (0.6‒1.3)	0.883
**Sarcopenia (SARC-F)**									
No	60 (57.7)	1		43(62.3)	1		54 (80.6)	1	
Yes	44 (42.3)	1.37 (1.1‒1.6)	**0.003**	26 (37.7)	1.1 1(0.7‒1.5)	0.540	13 (19.4)	1.10 (0.8‒1.4)	0.521
**Handgrip strength**									
Normal	73 (79.4)	1		46 (73.0)	1		48 (94.1)	1	
Low	19 (20.6)	1.17 (0.9‒1.5)	0.221	17 (27.0)	1.49 (1.0‒2.1)	**0.027**	3 (5.9)	1.06 (0.5‒1.9)	0.840
**Severe fatigue**									
No	25 (24.0)	1		11 (15.9)	1		12 (17.9)	1	
Yes	79 (76.0)	0.7 (0.6‒0.9)	**0.010**	58 (84.1)	1.23 (0.7‒2.0)	0.415	55 (82.1)	0.79 (0.6‒1.0)	0.082
**Pulmonary function**									
No comprimesed	24 (27.3)	1		15 (25.9)	1		14 (51.8)	1	
Compromised	64 (72.7)	1.22 (0.9‒1.6)	0.164	43 (74.1)	1.3 (0.8‒2.0)	0.255	13 (48.2)	1.35 (0.7‒2.4)	0.305
**Cognition**									
Normal	77 (75.5)	1		56 (82.4)	1		52 (88.1)	1	
Impaired	25 (24.5)	0.90 (0.6‒1.1)	0.491	12 (17.6)	0.67 (0.4 ‒1.1)	0.118	7 (11.9)	0.93 (0.7‒1.4)	0.765
**Reasons for physical therapy indication**									
LL weakness	38 (36.5)	1.71 (1.4‒2.0)	**0.000**	18 (26.1)	0.95 (0.6‒1.4)	0.812	-	-	-
UL weakness	23 (22.1)	1.62 (1.4‒1.8)	**0.000**	14 (20.3)	1.38 (0.9‒2.0)	0.098	-	-	-
Fatigue	29 (27.9)	1.61 (1.3‒1.8)	**0.000**	11 (15.9)	0.71 (0.4‒1.1)	0.199	-	-	-
Pain	13 (12.5)	1.43 (1.1‒1.7)	**0.000**	5 (7.3)	0.74 (0.3‒1.5)	0.433	-	-	-
Dyspnea	35 (33.7)	1.67 (1.4‒1.9)	**0.000**	18 (26.1)	1.09 (0.7‒1.6)	0.635	-	-	-
Sensitivity	4 (3.9)	1.2 (0.7‒1.8)	0.431	3 (4.3)	1.3 (0.6‒2.7)	0.487	-	-	-
Preconditions	29 27.9)	1.61 (1.3‒1.8)	**0.000**	21 (30.4)	1.72 (1.2‒2.3)	0.001	-	-	-

PR, prevalence ratio; n, number of patients; Cl, confidence interval; BADLs, basic activies of daily living; IADLs, instrumental activities of daily living; LL, lower limbs; UL, upper limbs. aPoisson.

Patients’ perception regarding why they were indicated for physical therapy 30 days post-discharge were: lower limb weakness (PR: 1.71; 95 %CI 1.4 - 2.0); dyspnea (PR: 1.67; 95 %CI 1.4 - 1.9); upper limb weakness (PR:1.62; 95 %CI 1.4 - 1.8); fatigue (PR: 1.61; 95 %CI 1.3 - 1.8); preconditions for Covid-19 (PR: 1.61; 95 %CI 1.3 - 1.8) and pain (PR: 1.43; 95 %CI 1.1 - 1.7) (Table 3).

However, those who were able to access physical therapy 30 days after discharge exhibited the greatest BADL limitations (PR: 1.81; 95 %CI 1.2 −2.6) and lower-than-expected handgrip strength (PR: 1.49; 95 %CI 1.0 - 2.1). The main reason cited was pre-existing conditions prior to Covid-19 (PR: 1.72; 95 %CI 1.2 - 2.3) (Table 3).

One year after discharge, the greatest access to physical therapy was observed in patients with moderate (PR: 1.63; 95 %CI 1.2–2.2), and high (PR: 1.65; 95 %CI 1.2 - 2.2) BADL limitations (PR: 1.70; 95 %CI 1.2–2.3), as well as concern about falls and dependence when walking (PR: 1.56; 95 %CI 1.1 - 2.1) (Table 3).

## Discussion

Based on the results obtained, 67 % of severely and critically ill patients who required hospitalization due to Covid-19 were indicated for physical therapy after discharge. However, the majority (53 %) were unable to access it within 30 days of discharge, taking up to one year to receive this care. Some patients exhibited functional impairment but were not recommended for post-discharge physical therapy. Receiving a physical therapy indication was significant, with a 4.07 and 2.06 likelihood of timely access to the service 30 days and one year after discharge, respectively. Timely and comprehensive continuity of care requires that patients receive a counter-referral to primary health care at discharge to ensure better coordination by health and physiotherapy services and provide more efficient treatment and use of resources^29^^,^^30^

The literature demonstrates the need for post-discharge rehabilitation due to functional sequelae resulting from the disease and hospitalization. However, there is no clear pathway for accessing health services^7^^,^^31^, ^32^, ^33^ Almeida et al. (2023)^34^ described patients' perception of a so-called "care gap" between hospitals and follow-up services, highlighting the fragility of the rehabilitation indication and post-discharge access process.

Problems caused by the Covid-19 pandemic include difficulty receiving a physical therapy indication and accessing the service after discharge. Although the literature suggests different scales for assessing respiratory dysfunction, muscle strength, balance, mobility, dyspnea, and fatigue at hospital discharge and recommendations for rehabilitation[35, 36, 37, 38] due to sequelae from hospitalization for Covid-19, it falls to healthcare professionals to indicate physiotherapy and provide recomendations on how and where to access treatment.

Our study shows that both functional impacts and signs and symptoms resulting from Covid-19 were clear reasons for recommending post-discharge physical therapy, with PR ranging from 0.07 to 1.71. However, there were few criteria with regard to accessing physical therapy.

Covid-19 infection, hospitalization, and ICU admission can lead to functional loss, disability, and ADL limitations,^2^^,^^7^^,^^8^ which is corroborated by our findings.

Of the functional impacts, BADL impairment was related to physical therapy indication and access 30 days and one year after discharge. To date, there is no established threshold for ADL limitations to recommend physical therapy. However, physiotherapy is known to promote functional independence. Research with this population has identified ADL limitations that can persist up to 6 months after discharge for hospitalization due to Covid-19^39^ These limitations demonstrate the need for post-discharge rehabilitation since they affect the quality of life of individuals^40^ Otoala et al. (2023)^41^ observed favorable functional evolution at the 6-month follow-up. However, 22 % of patients exhibited some degree of persistent frailty six months after discharge.

Post-Covid-19 functional limitation was related to physical therapy indication, with 87 % of patients displaying functional impairment after hospitalization for Covid-19. This corroborates literature findings, whereby most hospitalized patients have some degree of functional limitation according to the PCFS scale^42^ This scale evaluates the extent to which post-Covid-19 functional status is altered by disease sequelae and length of hospital stay, affecting quality of life and independence.

Fatigue was also a criterion for indicating physical therapy, with 76 % of patients exhibiting severe fatigue after discharge. Our findings are similar to those of Otoala et al. (2023), who reported fatigue in 69 % of participants 3 months after discharge^41^ Fatigue impacts ADLs, social activities, and mood.

Handgrip strength is an indicator of global strength and, in the present study, was a criterion for physical therapy access 30 days post-discharge, with decreased handgrip strength in 27 % of patients. This result is similar to that of Qorolli et al. (2023), who reported lower handgrip strength in 33 % of the study sample^43^

The most frequent reason for indicating continued physical therapy was lower limb weakness (36.5 %), followed by dyspnea, fatigue, pre-Covid-19 conditions, and pain, possibly due to functional limitations resulting from these symptoms. The majority (67 %) of participants in our study received some form of physiotherapy recommendation, but this was based on the subjective assessment of individual professionals and physical therapy indication was not proportional to access. Araya-Quintanilla et al. (2023) conducted a literature review on rehabilitation recommendations and effects and the main post-Covid-19 symptoms, with a greater likelihood of these persisting after hospitalization, but without referencing criteria for indicating continued physical therapy. They also highlighted the positive effects of rehabilitation programs with a multidisciplinary team, including physiotherapy, on recovering functional capacity and quality of life^44^

As such, it is important to consider functional assessment combined with signs and symptoms as a parameter or criterion for physical therapy indication, in addition to the hospital discharge report and clinical evaluation, in order to ensure timely and effective recommendations and access to care. This will also enable better coordination in healthcare networks for physical therapy care continuity.

It is important to note that, in addition to the configuration and structure of the health system to address care needs, the pandemic also contributed to access difficulties. Hospital and specialist care were considered priorities for regular follow-up of Covid-19 health problems^45^^,^^46^

In the present study, of the 47 % of the patients who had access to physical therapy in the first 30 days after discharge, the service was largely provided at the public hospital where they were hospitalized in the city of São Paulo (61 %), followed by outpatient clinics/private hospitals (11 %), basic health units (9 %) and public outpatient clinics (9 %). After one year, 71 % of patients had access to physical therapy, 54 % of whom received it at the public hospital in São Paulo where the research was conducted, 32 % at SUS outpatient clinics and 5 % at Basic Health Units.

These findings are similar to those of Almeida et al. (2023), who reported that the vast majority of respondents were contacted by the hospital where they had been hospitalized for continued rehabilitation, followed by an active search for patients themselves, also highlighting access difficulties due to ack of knowledge about the care network on the part of professionals, and problems in post-discharge counter-referral flow. This resulted in patients abandoning treatment between hospital discharge and the beginning of rehabilitation, representing a rupture in care trajectories^34^

In this respect, we highlight the need to establish more specific functional criteria, signs, and symptoms for physical therapy indication at discharge as part of the dehospitalization process and to reduce the post-discharge "care vacuum" of critically ill patients. This strategy is important beyond Covid-19, since the care vacuum and challenges inherent to rehabilitation in primary and specialist services prompt users to seek private care. This compromises household income, exacerbates fragmentation and increases the direct search for focal specialists, weakening guaranteed access to physiotherapy. To avoidthis scernario, physiotherapy should be provided via primary health services to ensure comprehensive and coordinated care supported by specialized public service and rehabilitation networks, in line with the principles of humanized care^34^

Finally, it is important to underscore the scarcity of research regarding continuity of care with post-discharge physical therapy indication and access following hospitalization for Covid-19. Thus, our study contributes to bridging this gap by assessing the associations between Covid-19 functional impacts and physical therapy indication and access after discharge in severely and critically ill patients hospitalized for Covid-19.

### Strengths and limitations

The sample consisted of patients with Covid-19 admitted to a referral hospital for critically ill patients in the city of São Paulo, Brazil. No other study has analyzed Covid-19 functional impacts and continuity of physical therapy care post-discharge in critically ill patients.

The results obtained should be interpreted considering the following limitations: the study population was extracted from a convenience sample of critically ill patients during the pandemic and as such, the results are not generalizable to mild or moderate cases of Covid-19. Given the type of sample used, selected during the critical stage of the Covid-19 pandemic, some areas of the analysis may have been subject to selection bias, such as collider bias and missing data. Symptom severity, time until physiotherapy access and possible factors related to lack of access could not be assessed.

## Conclusion

Physical therapy was indicated for most of the severely and critically ill patients studied, but without timely access after hospital discharge. Despite the lack of functional criteria at discharge, patients with poor functional performance perceived the need for physical therapy indication and referral. However, an indication for continued physical therapy after discharge was essential for timely access to these services within the healthcare network. In light of the above, in addition to the use of functional criteria, it is recommended that physical therapy indications and counter-referrals be included in the hospital discharge report.

## Funding

The study was supported by grants 402,698/2020–0 and 312,279/2018–3 from the Conselho Nacional de Desenvolvimento Científico e Tecnológico (CNPq) and 19,618–8/2018 from the Sao Paulo State Research Foundation (FAPESP)."

## Declaration of competing interest

The authors declare no conflicts of interest.
